# HTLV-1 Infection and Rheumatic Diseases

**DOI:** 10.3389/fmicb.2020.00152

**Published:** 2020-02-11

**Authors:** Kunihiko Umekita, Akihiko Okayama

**Affiliations:** Department of Rheumatology, Infectious Diseases, and Laboratory Medicine, Faculty of Medicine, University of Miyazaki, Miyazaki, Japan

**Keywords:** human T-cell leukemia virus type 1, rheumatic diseases, rheumatoid arthritis, adult T-cell leukemia/lymphoma, HTLV-1-associated myelopathy/tropical spastic paraparesis, disease-modifying anti-rheumatic drugs

## Abstract

Some major research and clinical questions about human T-cell leukemia virus type 1 (HTLV-1) infection and rheumatic diseases remain: (1) Does HTLV-1 infection cause rheumatic diseases? (2) Do patients with rheumatic diseases display different responses to treatment with anti-rheumatic agents when they are HTLV-1 carriers? (3) Is adult T-cell leukemia/lymphoma (ATL) or HTLV-1-associated myelopathy/tropical spastic paraparesis (HAM/TSP) more prevalent in HTLV-1 carriers with rheumatic diseases who are treated with anti-rheumatic agents? These questions are important because increasing numbers of patients with rheumatic diseases are currently receiving treatment with aggressive medicines such as immunosuppressants and biologics. Studies on HTLV-1 gene-transgenic mice have shown manifestations resembling rheumatic diseases. Epidemiological studies have shown a high incidence of HTLV-1 infection in patients with rheumatic diseases including rheumatoid arthritis (RA), Sjogren’s syndrome, and polymyositis. HTLV-1-positive and HTLV-1-negative patients with RA have displayed similar immunological features including the seroprevalence of anti-citrullinated peptide antibodies. Conversely, attenuated effectiveness of tumor necrosis factor inhibitors for HTLV-1-positive patients with RA in Japan has been reported. Therefore, although no direct evidence has shown that HTLV-1 infection alone causes rheumatic diseases, HTLV-1 may affect the inflammation of RA. Although the incidence of ATL or HAM/TSP among patients with rheumatic diseases has not been investigated in large-scale studies, ATL or HAM/TSP has developed among HTLV-1-positive patients with rheumatic diseases. HTLV-1 infection may affect the clinical course of patients with rheumatic diseases, particularly after receiving anti-rheumatic agents. Because studies on these issues are limited, further investigation with large sample sizes is necessary.

## Introduction

Human T-cell leukemia virus type 1 (HTLV-1) is a human retrovirus that is a causative agent of adult T-cell leukemia/lymphoma (ATL) and HTLV-1-associated myelopathy/tropical spastic paraparesis (HAM/TSP) (Poiesz et al., 1980; Hinuma et al., 1981; Gessain et al., 1985; Osame et al., 1986). There have been several reports about the relationship between HTLV-1 infection and rheumatic diseases; however, the results remain inconclusive (Martin et al., 2014). Rheumatoid arthritis (RA) is the most common rheumatic disease, and it is defined as chronic inflammation of the joints, followed by their destruction. Genetic factors such as certain types of HLA-DR genes and polymorphisms of multiple genes are considered important for its etiology (Hill et al., 2003; Deane et al., 2017). Smoking, gingivitis, and Epstein–Barr virus (EBV) infection have been also considered environmental risk factors, particularly in relation to the production of autoantibodies (Balandraud and Roudier, 2018). To maintain the chronic inflammation of RA, cell–cell interactions and interactions with cytokines among lymphocytes, macrophages, and synovial cells in the joints play crucial roles. Recently, medicines including the targets of these cell–cell interaction and cytokines have been developed and have dramatically improved the clinical course of RA (Burmester and Pope, 2017).

The major research and clinical questions about HTLV-1 infection and rheumatic diseases evaluated in this article are as follows: (1) Does HTLV-1 infection cause rheumatic diseases? (2) Do patients with rheumatic diseases display a different response to treatment with anti-rheumatic agents or immunosuppressive agents when they are HTLV-1 carriers? (3) Are ATL or HAM/TSP more prevalent in HTLV-1 carriers with rheumatic diseases who are treated with anti-rheumatic agents? We reviewed the studies on these topics (mainly the relationship between HTLV-1 infection and RA) and described what we know and what areas of HTLV-1 research must be addressed in the future.

## HTLV-1 and Rheumatic Diseases

It remains unclear whether HTLV-I infection etiologically contributes to the development of RA. Several epidemiological studies have shown that the prevalence of HTLV-1 infection is higher in patients with rheumatic diseases such as RA, polymyositis, and Sjogren’s syndrome than that in healthy controls such as blood donors (Morgan et al., 1989; Eguchi et al., 1992, 1996). A systematic review of epidemiological studies showed that HTLV-1 infection is associated with increased risk of RA and Sjogren’s syndrome among rheumatic diseases (Schierhout et al., 2019). However, a limited number of reports exist, and they are inconclusive regarding whether HTLV-1 infection is more prevalent in patients with rheumatic diseases than that in the general population. Chronic inflammatory diseases including arthritis have been shown to develop in transgenic mice with the HTLV-1 *Tax* and *HBZ* genes (Iwakura et al., 1995; Satou et al., 2011). A certain proportion of HTLV-1-positive patients with arthritis have been reported to display mono- or oligo-arthritis of the large joints (Sato et al., 1991). Biopsy samples from their synovial tissues tested positive for HTLV-1. In the 1990s, the concept of HTLV-1-associated arthropathy (HAAP) was proposed (Kitajima et al., 1991), although it remains unclear whether HAAP differs from HTLV-1-positive RA.

An exocrinopathy resembling Sjogren’s syndrome was reported in HTLV-1 *tax* transgenic mice (Green et al., 1989). Compared with HTLV-1-negative patients, HTLV-1-positive patients with Sjogren’s syndrome were reported to have a higher prevalence of uveitis and lung diseases but lesser anti-nuclear antibodies (Nakamura et al., 2015). These characteristics are more evident in HTLV-1-positive patients with Sjogren’s syndrome, which is associated with HAM/TSP. These findings suggest the relationship between these diseases.

These results may suggest the effect of HTLV-1 infection in the etiology of rheumatic diseases; however, HTLV-1-positive patients comprise only a minor proportion of patients with rheumatic diseases, even in the most prevalent areas of HTLV-1. HTLV-1-positive patients comprised only 6% of patients with RA in our cohort in Miyazaki, Japan, which is one of the most endemic areas for HTLV-1 (Umekita et al., 2019). The clinical features and laboratory data including the prevalence of rheumatoid factor and anti-cyclic citrullinated peptide antibodies are similar between HTLV-1-positive and HTLV-1-negative patients (Umekita et al., 2019). The similarity of clinical features and laboratory data between HTLV-1-positive and HTLV-1-negative patients has also been observed in other cohorts (Suzuki et al., 2018). Therefore, it is difficult to conclude that HTLV-1 infection alone causes RA. However, it is still being determined whether HTLV-1 infection is a causative agent for arthropathy or polyarthritis, especially when the patients are seronegative for these autoantibodies.

Conversely, HTLV-1 primarily infects CD4 + T-lymphocytes and is considered to alter their functions and lineages. Certain clones of HTLV-1-infected cells proliferate and cause the development of ATL after malignant transformation. Most ATL cells are CD25 + CCR4 + and express high levels of FoxP3, which is a hallmark of regulatory T-cells (Kannagi et al., 2019). Elevated levels of IL-10 in the serum are reported in patients with ATL and are considered to be related to the immunosuppressive condition.

By contrast, HAM/TSP is a chronic inflammatory disease of the central nervous system that displays high levels of HTLV-1 proviral load (PVL) and polyclonal expansion of HTLV-1-infected cells. Peripheral blood mononuclear cells isolated from patients with HAM/TSP showed autonomously produced inflammatory cytokines such as interferon (IFN)-gamma, IL-6, and TNF-alpha (Tendler et al., 1991). HTLV-1 Tax was reported to be one of the activators of nuclear factor kappa-light-chain-enhancer of activated B cells. In addition, HTLV-1 Tax was shown to activate the *t-bet* gene with reduced expression of FoxP3 in the infected cells, resulting in their differentiation toward Th1 in HAM/TSP (Yamano et al., 2009; Yamamoto-Taguchi et al., 2013; Araya et al., 2014). Chemokine production increased in cultured peripheral blood mononuclear cells obtained from patients with HAM/TSP (Montanheiro et al., 2007). CD4 + CD25 + CCR4 + T-lymphocytes in HAM/TSP produce IFN-gamma, activate astrocytes in the central nervous system with CXCL10 expression, and induce the migration of Th1-like T-lymphocytes into the central nervous system (Ando et al., 2013). This positive-feedback loop is hypothesized to be related to the progression of HAM/TSP. Both HTLV-1 carriers and patients with HAM/TSP have been reported to be associated with various chronic inflammatory diseases including rheumatic diseases (Nakamura et al., 1997; Furuya et al., 1998; Murphy et al., 2004; Yakova et al., 2005).

The number of HTLV-1-infected cells has been reported to increase not only in the peripheral blood but also in the synovial fluid of patients with RA (Yakova et al., 2005), although the roles of these HTLV-1-infected cells in the pathogenesis of RA has not yet been revealed. *Ex vivo* cultures of lymphocytes from HTLV-1 carriers show spontaneous proliferation (Prince et al., 1991). This was also observed more evidently in patients with HAM/TSP (Itoyama et al., 1988; Eiraku et al., 1992). The production of TNF and IFN-gamma was observed in cultured peripheral blood mononuclear cells obtained from patients with HAM/TSP (Montanheiro et al., 2009; Araya et al., 2014). If a process, similar to that of HAM/TSP, occurs in HTLV-1-positive patients with RA, HTLV-1 infection can be an environmental factor responsible for the initiation and/or maintenance of chronic inflammation in rheumatic diseases. However, further clarification is necessary to determine whether HTLV-1-infected T-lymphocytes in patients with RA show characteristics that resemble HTLV-1-infected cells in ATL or HAM/TSP. Dysregulation in the balance between functionally opposite cytokines such as IFN-gamma/IL-10 balance are considered to contribute to the pathogenesis of HTLV-1 infection (Futsch et al., 2018). Thus, an analysis of the function of HTLV-1-infected T-lymphocytes and cytokines in rheumatic diseases is necessary.

## Response to the Anti-Rheumatic Treatments of HTLV-1-Positive Patients with RA

HTLV-1-positive patients with RA showed higher levels of C-reactive protein, which is a marker for RA activity, in the peripheral blood before receiving treatment with disease-modifying anti-rheumatic drugs (DMARDs) compared with HTLV-1-negative patients (Umekita et al., 2014). Two retrospective observational studies showed the attenuated effectiveness of TNF inhibitors in HTLV-1-positive patients with RA (Umekita et al., 2014; Suzuki et al., 2018). These data suggested that HTLV-1 infection induces more inflammation of RA and contributes to the attenuated effectiveness of TNF inhibitors, although its mechanism is not clear. Another important question is determining whether HTLV-1-positive patients with RA have resistance not only to TNF inhibitors but also to other DMARDs.

## Development of ATL and HAM/TSP in HTLV-1-Positive Patients with RA

It is unknown whether the association of rheumatic diseases and treatment against such diseases affects the natural history of HTLV-1 infection, particularly in view of the development of HTLV-1-associated diseases such as ATL and HAM/TSP.

Patients with RA have reported higher incidence rates of malignant lymphoma, although the surface phenotype of lymphoma found in RA is B-cell predominant (Klein et al., 2018). For ATL, a higher PVL, advanced age, family history of ATL, and first opportunity for HTLV-1 testing during treatment for other diseases were reported as independent risk factors for the progression of ATL in HTLV-1 carriers (Iwanaga et al., 2010). The HTLV-1 PVL in the peripheral blood of patients with RA and patients with other rheumatic diseases was reported to be higher than that in matched asymptomatic HTLV-1 carriers (Yakova et al., 2005). These reports suggest the possibility that HTLV-1-positive patients with RA may have a high risk for ATL development.

An additional important factor that must be recognized for the risk for ATL in HTLV-1-positive RA patients is the impact of DMARD treatment on RA. The representative DMARD, methotrexate (MTX), is well known as the anchor drug for RA with immunosuppressive effects. Another DMARD, tacrolimus, specifically suppresses T-cell function by inhibiting calcineurin. Several biological DMARDs mainly inhibit the function of proinflammatory cytokines such as TNF and IL-6. Recently, novel DMARDs were developed, which inhibit Janus kinase for signal transduction from the receptors of proinflammatory cytokines. It is unknown whether there are any direct effects of these medicines on HTLV-1-infected cells *in vivo*; however, it is clear that these medicines cause various levels of immune suppression. HTLV-1 carriers with mother-to-child infection were shown to have higher HTLV-1 PVLs in the peripheral blood than those with infection between spouses (Ueno et al., 2012). The clonal expansion of HTLV-1-infected T-lymphocytes in HTLV-1 carriers was considered to be controlled by the pressure of an immune surveillance system for long time (Tanaka et al., 2005). A clone developed to ATL was reported to exist in the peripheral blood even 8 years before the onset of ATL (Okayama et al., 2004). An HTLV-1 Tax-specific cytotoxic T-lymphocyte response has been reported in asymptomatic HTLV-1 carriers (Takamori et al., 2011). The favorable outcomes of the vaccination treatment in combination with HTLV-1 Tax and dendritic cells in patients with ATL, who experienced hematopoietic stem cell transplantation, suggests the importance of immune system surveillance for preventing the development of ATL (Suehiro et al., 2015). Therefore, there is a possibility that immunosuppression caused by DMARDs promotes the development of ATL, particularly if a patient with RA already has the clones of HTLV-1-infected cells with a nature similar to ATL cells. Because no data are available on the analysis of clonal expansion of HTLV-1 infected cells in patients with RA, further studies are necessary in the future.

In addition, the incidence of lymphoproliferative disorders (LPDs) including lymphoma has been reported to increase in RA patients who were treated with MTX (Ichikawa et al., 2013). LPDs that are related to treatment with immunosuppressive drugs are categorized as “other iatrogenic immunodeficiency-associated LPDs” (Tokuhira et al., 2019). Most of the LPD cases reported are B-cell type or Hodgkin’s disease, and the involvement of EBV infection in these LPD cases is suspected; however, several cases of ATL have been reported in patients with rheumatic diseases during treatment with DMARDs including MTX (Supplementary Table S1; Fujiwara et al., 2006; Bittencourt et al., 2013; Nakamura et al., 2013; Hashiba et al., 2018; Takajo et al., 2018; Okamoto et al., 2019). MTX-EVB-related LPDs often show spontaneous regression after the cessation of MTX (Ichikawa et al., 2013). This phenomenon was also reported in cases of ATL in RA patients treated with MTX (Hashiba et al., 2018; Takajo et al., 2018). A high incidence of ATL in liver transplantation recipients who received tacrolimus as an immunosuppressant was also reported (Kawano et al., 2006). Conversely, reports observing HTLV-1-positive RA patients for several years showed that ATL cases are rarely identified (Umekita et al., 2015, 2019; Eguchi et al., 2019). Treatment with DMARDs including both MTX and biologics did not significantly increase HTLV-1 PVLs in HTLV-1-positive patients with RA (Umekita et al., 2019). However, the cessation of MTX treatment decreased HTLV-1 PVLs in HTLV-1-positive RA patients with a high PVL in the same report (Umekita et al., 2019). Therefore, it remains uncertain whether treatment with DMARDs contributes to the development of ATL in HTLV-1-positive RA patients.

It is important to recognize that two major routes exist in natural HTLV-1 infection: mother-to-child transmission in infancy and transmission between spouses in adulthood (Kajiyama et al., 1986; Sugiyama et al., 1986; Iga et al., 2002; Hino, 2011). HTLV-1 carriers including patients with RA are a heterogeneous population from the point of view of infectious routes. The incidence of ATL among HTLV-1 carriers who are infected in adulthood is very low (Murphy et al., 1989); however, HAM/TSP has been reported to occur in HTLV-1 carriers who acquired infection in adulthood via blood transfusion and transmission between spouses (Sakai et al., 1989; Krämer et al., 1995). HTLV-1 infection occurs more predominantly from husband to wife (Kajiyama et al., 1986; Iga et al., 2002). More than 4,000 female predominant HTLV-1 infections a year in adults were estimated to occur in Japan based on blood donation data (Satake et al., 2016). Because 80% of RA patients are female, HTLV-1 infection in patients with RA between spouses is not considered a rare event. Therefore, it is important to determine whether not only ATL but also HAM/TSP develops in HTLV-1-positive patients with RA.

Immune status in patients with RA is considered Th1 and Th17 dominant. The immune status of HAM/TSP is also considered Th1 dominant. Therefore, whether RA affects the etiology and clinical condition of HAM/TSP is an important question. We must also consider the effect of the DMARDs that patients with RA are receiving. Indeed, worsening symptoms of both HAM/TSP and HTLV-1-associated uveitis were reported in an HTLV-1-positive patient with RA who received treatment with a soluble IL-6 receptor inhibitor (Terada et al., 2017). More recently, a high incidence of HAM/TSP in renal transplantation recipients from HTLV-1-positive donors was reported (Yamauchi et al., 2019). These data suggest that the immune status of the host may affect the development of HAM/TSP in HTLV-1 carriers. In this respect, we need more data on HTLV-1-positive patients with rheumatic diseases and their treatment. It is also an important question of whether the incidence of HAM/TSP in a patient with RA, who has been an HTLV-1 carrier or who acquired HTLV-1 infection in adulthood, is higher than that in asymptomatic carriers. Indeed, the prevalence of comorbidities such as Sjogren’s syndrome and RA in patients with HAM/TSP is recently reported to be as high as 3.7 and 2.7%, respectively (Tsutsumi et al., 2019). This prevalence in patients with HAM/TSP appears to be higher than that in the general population. Because anti-inflammatory agents such as corticosteroids are used in the management of HAM/TSP (Nozuma and Jacobson, 2019), we must consider whether these agents affect the clinical presentation of rheumatic diseases.

## Discussion

**FIGURE 1:**
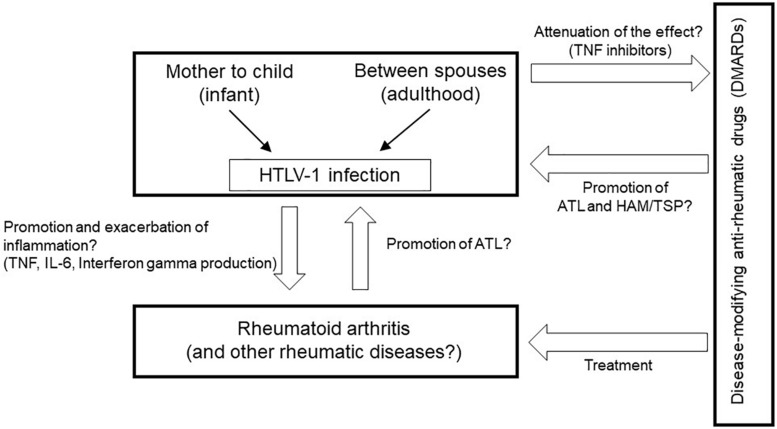
Clinical and research questions about the relationships among rheumatic diseases, treatment using immune-modifying medicines, and HTLV-1 infection.

It is important to understand the relationships among rheumatic diseases, treatment using immune modulating or immunosuppressive drugs, and chronic viral infection such as HTLV-1 (Figure 1); however, it is clear that studies about these issues have been very limited thus far. Moreover, almost no data are available on rare but representative rheumatic diseases such as systemic lupus erythematosus, which we could not mention in this article.

Currently, the HTLV-1 screening test conducted in pregnant women for preventing HTLV-1 transmission to their child has been performed all over the country of Japan. Consequently, most pregnant women know if they are positive for HTLV-1. Because rheumatic diseases such as RA are generally more common in females than in males, more patients with rheumatic diseases and their doctors are assumed to be anxious about the effects of HTLV-1 infection on the disease condition and treatment using DMARDs than in the past. We previously performed a nationwide investigation in Japan using a questionnaire for rheumatologists that addressed these issues. Indeed, many doctors mentioned that they did not have enough knowledge about HTLV-1 infection and claimed the necessity of having guidelines on how to treat HTLV-1-positive patients with RA.

The clinical presentation of ATL and HAM/TSP is different between Japanese and Caribbean populations. The onset age of ATL in the Caribbean population is earlier than that in the Japanese population (Hanchard, 1996). The frequency to develop HAM/TSP among HTLV-1 carriers in populations from Caribbean and African origins in England is higher than that in the Japanese population (Tosswill et al., 2000). Advanced treatment for rheumatic diseases is common in many countries. Therefore, the questions raised in this article are not only issues in Japan and it is necessary to obtain global data regarding HTLV-1 infection in rheumatic diseases. We must clarify whether doctors should pay special attention when a patient with rheumatic disease is positive for HTLV-1. It is also important to determine whether rheumatologists should test for HTLV-1 before beginning treatment using DMARDs. In other words, it raises the question of whether DMARDs should be prescribed to patients who are aware of their HTLV-1 status. Further large-scale investigations about HTLV-1-positive patients with rheumatic diseases and a long observation period are necessary.

## Author Contributions

All authors listed have made a substantial, direct and intellectual contribution to the work, and approved it for publication.

## Conflict of Interest

The authors declare that the research was conducted in the absence of any commercial or financial relationships that could be construed as a potential conflict of interest.
