# Supplementary material for: HTLV-1 Infection and Rheumatic Diseases
Source: Front Microbiol. 2020 Feb 11;11:152. doi: 10.3389/fmicb.2020.00152 (PMC7025999; doi:10.3389/fmicb.2020.00152)
Supplement: Supplementary file 1 [file Data_Sheet_1.PDF]

Table Literature review: Development of ATL in patients with rheumatic diseases

Authors	Age	Sex	Rheumatic disorders	Duration	DMARDs (duration of MTX treatment)	Type of ATL	sIL-2R (U/mL)	Therapeutic regimen for ATL	ATL outcome
Hashiba Y, et al	78	F	RA	8	MTX (for 6 years)<br>Infliximab<br>Corticosteroid	Chronic	2,400	Withdrawal<br>MTX, infliximab	Remission
Takajo I, et al	75	F	RA	5	MTX (for 5 years)<br>Corticosteroid	Lymphoma	34,881<br>↓<br>663<br>↓<br>73,208	Withdrawal MTX	Improve<br>↓<br>Flare, death
Okamoto M, et al	70	F	RA+IP	22	Cyclophosphamide<br>Abatacept (ABT)<br>Corticosteroid<br>Iguratimod (IGU)	Lymphoma	11,066	Withdrawal<br>ABT and IGU<br>↓<br>Chemotherapy	Death
Okamoto M, et al	71	M	RA	4	MTX (for 2 years)<br>Corticosteroid	Acute	No data	Withdrawal MTX<br>↓<br>Chemotherapy	Improve<br>↓<br>Flare, death
Nakamura H, et al	50	F	RA	11	MTX (for 4 + 2 years)<br>Tocilizumab<br>Corticosteroid	Chronic<br>↓<br>Acute	11,000	Withdrawal<br>MTX and tocilizumab<br>↓<br>Chemotherapy + BMT	Remission
Bittencourt AL, et al	48	F	AS	Not Available	Adalimumab	Chronic	No data	Withdrawal adalimumab<br>↓<br>IFNα + Zidovudine	Remission
Fujiwara H, et al	66	F	MCTD	1	Corticosteroid	Acute	162,000	Chemotherapy	Death

DMARDs: disease-modifying anti-rheumatic drugs, ATL: adult T-cell leukemia, sIL-2R: soluble interleukin-2 receptor, RA: rheumatoid arthritis, MTX: methotrexate, BMT: bone marrow transplantation, AS: ankylosing spondylitis, IFN: interferon, MCTD: mixed connective tissue disease
